# Cardiomyocyte PIK3R1 deficiency attenuates mitochondrial dynamic imbalance and ferroptosis in diabetic cardiomyopathy in association with the PI3K/Akt/Nrf2 axis

**DOI:** 10.3389/fcell.2026.1953037

**Published:** 2026-10-21

**Authors:** Jiefang Zhou, Qianqian Xiao, Fangfang Zhang, Xiuwei Shen, Shujuan Wu, Linling Wang, Chunyan Huang

**Affiliations:** 1 Department of Clinical Pharmacy, Shaoxing TCM Hospital Affiliated to Zhejiang Chinese Medical University, Shaoxing, China; 2 Department of Clinical Pharmacy, The People’s Hospital of Pingyang, Wenzhou, China; 3 Department of Clinical Pharmacy, The Third Affiliated Hospital of Wenzhou Medical University, Wenzhou, China; 4 Department of Pharmacy, The Third Affiliated Hospital of Wenzhou Medical University, Wenzhou, China; 5 Department of Medicine Pharmacy, Shaoxing TCM Hospital Affiliated to Zhejiang Chinese Medical University, Shaoxing, China

**Keywords:** cardiomyocyte, diabetic cardiomyopathy, ferroptosis, mitochondrial homeostasis, PIK3R1

## Abstract

**Objective:**

PIK3R1 encodes the p85α regulatory subunit of phosphatidylinositol 3-kinase (PI3K) and negatively regulates PI3K signaling. Our prior study identified abnormal PIK3R1 expression in diabetic cardiomyopathy (DCM), but its role and underlying mechanisms in DCM pathogenesis remain unclear. This study aimed to elucidate the functional significance of PIK3R1 and its molecular mechanism in regulating mitochondrial homeostasis, cardiomyocyte ferroptosis, and DCM progression.

**Methods:**

A DCM mouse model was established by high-fat diet (HFD) feeding combined with low-dose streptozotocin (STZ) injection. Cardiomyocyte-specific PIK3R1 knockout (PIK3R1KO) mice were generated using the Cre-LoxP system. Five *in vivo* cohorts were studied: control and DCM mice; DCM mice treated with the ferroptosis inhibitor ferrostatin-1; DCM mice treated with the mitochondrial fission inhibitor Mdivi-1; wild-type and PIK3R1KO mice with or without the PI3K/Akt agonist 740 Y-P; and PIK3R1KO DCM mice receiving AAV9-Drp1 or AAV9 vector. Cardiac function was evaluated by echocardiography. Mitochondrial morphology and function were assessed by transmission electron microscopy, myocardial ATP content, mitochondrial DNA copy number and reactive oxygen species (ROS) detection. Cardiomyocyte ferroptosis was determined by iron content measurement, lipid peroxidation assay, and expression analysis of ferroptosis-related markers (GPX4, ACSL4, SLC7A11). The PI3K/Akt/Nrf2 signaling pathway was examined using Western blot and immunofluorescence staining.

**Results:**

DCM mice exhibited impaired cardiac function (decreased left ventricular ejection fraction and fractional shortening), myocardial apoptosis, mitochondrial dysfunction (fragmentation, reduced ATP content, increased ROS production), and enhanced ferroptosis (elevated iron deposition, lipid peroxidation, downregulated GPX4 and SLC7A11, upregulated ACSL4). Treatment with the mitochondrial division inhibitor Mdivi-1 improved mitochondrial homeostasis, inhibited ferroptosis, and attenuated DCM progression. PIK3R1 deficiency in DCM mice significantly ameliorated mitochondrial dynamic imbalance, suppressed cardiomyocyte ferroptosis, and rescued cardiac function by activating the PI3K/Akt/Nrf2 pathway. However, Drp1 overexpression reversed the protective effects of PIK3R1 knockout on mitochondrial function and ferroptosis in DCM mice.

**Conclusion:**

Cardiomyocyte PIK3R1 deficiency is associated with preserved mitochondrial dynamic homeostasis, reduced cardiomyocyte ferroptosis and attenuated DCM progression, together with increased PI3K/Akt/Nrf2 signalling. Whether this pathway mediates the effect requires direct loss-of-function testing, and PIK3R1 may warrant evaluation as a candidate therapeutic target for DCM.

## 1 Introduction

Diabetic cardiomyopathy (DCM) is a major complication of diabetes mellitus, characterized by myocardial structural and functional abnormalities independent of coronary artery disease, hypertension, or other cardiovascular risk factors (Zhang and Chen, 2012). The pathogenesis of DCM involves multiple mechanisms, including oxidative stress, mitochondrial dysfunction, cardiomyocyte apoptosis, and metabolic disorders (Capece et al., 2026). Recent studies have highlighted ferroptosis, an iron-dependent regulated cell death driven by lipid peroxidation, as a key contributor to DCM progression (Dixon et al., 2012). However, the molecular mechanisms underlying ferroptosis in DCM remain incompletely understood.

PIK3R1, located on human chromosome 5q13.1, encodes the p85α regulatory subunit of PI3K, which plays a critical role in regulating PI3K/Akt signaling (Chen et al., 2019). The PI3K/Akt pathway is a key intracellular signaling cascade involved in cell survival, proliferation, metabolism, and mitochondrial function (Manning and Cantley, 2007). PIK3R1 negatively regulates PI3K activity by binding to the catalytic subunit p110, thereby inhibiting Akt phosphorylation and downstream signaling (Fruman and Rommel, 2014). Abnormal expression of PIK3R1 has been implicated in various cardiovascular diseases, including heart failure, myocardial infarction, and arrhythmia (Liu et al., 2026; van Deel et al., 2025). Our previous bioinformatics analysis identified PIK3R1 as a differentially expressed gene in DCM, but its specific role and mechanism in DCM remain unclear.

Mitochondrial homeostasis, including mitochondrial biogenesis, fission, fusion, and mitophagy, is essential for maintaining cardiac function (Hinton et al., 2024). Dysfunction of mitochondrial dynamics is closely associated with cardiomyocyte injury and DCM progression (Gollmer et al., 2020). The nuclear factor erythroid 2-related factor 2 (Nrf2) is a master regulator of antioxidant defense, which can activate the transcription of downstream target genes such as glutathione peroxidase 4 (GPX4) to inhibit lipid peroxidation and ferroptosis (Jiang et al., 2024). Emerging evidence suggests that the PI3K/Akt/Nrf2 pathway is involved in the regulation of mitochondrial homeostasis and ferroptosis (Yang et al., 2025). Whether cardiomyocyte PIK3R1 is linked to mitochondrial dynamic imbalance and ferroptosis in DCM, and whether PI3K/Akt/Nrf2 signalling accompanies that link, has not been examined.

In this study, we established a DCM mouse model and generated cardiomyocyte-specific PIK3R1 knockout mice to explore the role of PIK3R1 in DCM. We tested the hypothesis that cardiomyocyte PIK3R1 contributes to mitochondrial dynamic imbalance and ferroptosis in DCM, and we examined PI3K/Akt/Nrf2 signalling as a candidate mediator of that contribution, recognising that the present design addresses the former by loss of function and the latter by association only. Our findings provide new insights into the molecular mechanism of DCM and identify PIK3R1 as a potential therapeutic target for DCM.

## 2 Materials and methods

### 2.1 Animals and experimental groups

C57BL/6 J mice (male, 6–8 weeks old) were purchased from Shanghai Slack Laboratory Animal Co., Ltd (Shanghai, China). Cardiomyocyte-specific PIK3R1 knockout (PIK3R1KO) mice were generated by crossing PIK3R1 flox/flox mice (Jackson Laboratory, United States) with α-MHC-Cre transgenic mice (Jackson Laboratory, United States), both of which are established and previously characterised lines. The genotype of every animal was confirmed before entry into the study by PCR of tail-tip genomic DNA for the floxed Pik3r1 allele and for the Cre transgene, and Cre-negative flox/flox littermates were used as wild-type (WT) controls throughout. All mice were housed in a specific pathogen-free (SPF) environment with a 12-h light/dark cycle, and provided with food and water *ad libitum*. Only male mice were used, because female mice show variable susceptibility to streptozotocin-induced hyperglycaemia and because oestrogen is cardioprotective in this model; a single sex was therefore studied to limit variability in model induction.

The DCM model was established by high-fat diet (HFD, 60% fat content, Research Diets, United States) feeding from week 0, followed at week 8 by intraperitoneal injection of low-dose streptozotocin (STZ, 35 mg/kg/day, Sigma-Aldrich, United States) dissolved in 50 mM sodium citrate buffer (pH 4.5) on three consecutive days; control mice received a normal diet (ND) and an equal volume of citrate buffer. HFD feeding was maintained until the end of the study. Mice with fasting blood glucose ≥11.1 mmol/L and random blood glucose ≥16.7 mmol/L at 2 weeks after the last STZ injection were considered successful DCM models. Fasting blood glucose was measured monthly and body weight every 2 weeks. All mice were followed to week 24, when echocardiography, blood sampling and heart harvest were performed; no animals were maintained beyond week 24 and no interim sacrifice cohorts were used.

Mice were randomly allocated to the following cohorts, with 10 animals per group.

Cohort 1 (model characterisation): CON, C57BL/6 J mice fed ND and given citrate buffer; DCM, C57BL/6 J mice subjected to HFD and STZ.

Cohort 2 (ferroptosis inhibition): CON; DCM; and Fer-1, DCM mice given ferrostatin-1 (2 mg/kg, MedChemExpress, United States) intraperitoneally every other day during the last 2 weeks of the study (weeks 22–24). The CON and DCM groups received an equal volume of PBS by the same route and schedule.

Cohort 3 (mitochondrial fission inhibition): CON; DCM; and Mdivi-1, DCM mice given Mdivi-1 (50 mg/kg/day, Sigma-Aldrich, United States) intraperitoneally for 2 weeks (weeks 22–24). The CON and DCM groups received an equal volume of PBS by the same route and schedule.

Cohort 4 (genotype and pathway activation): WT-CON, WT-DCM and WT-740 Y-P, and the corresponding PIK3R1KO-CON, PIK3R1KO-DCM and PIK3R1KO-740 Y-P groups. 740 Y-P (10 mg/kg, MedChemExpress, United States) was given intravenously every other day during weeks 22–24; the CON and DCM groups received an equal volume of PBS by the same route and schedule.

Cohort 5 (Drp1 rescue): WT-CON, WT-DCM, PIK3R1KO-CON, PIK3R1KO-DCM, PIK3R1KO-AAV-Drp1 and PIK3R1KO-AAV-NC. Adeno-associated virus serotype 9 carrying Drp1 (AAV9-Drp1) or the corresponding empty vector (AAV9-NC) (GeneChem, China) was injected via the tail vein at week 12, that is 4 weeks after STZ.

The experimental protocol was approved by the Animal Ethics Committee of Third Affiliated Hospital of Wenzhou Medical University in March 2025 (RY20250319). All animal experiments were performed in accordance with the Guide for the Care and Use of Laboratory Animals (NIH Publication No. 85-23, revised 2011).

### 2.2 Echocardiographic assessment

Cardiac function was evaluated by echocardiography using a Vevo 2100 system (VisualSonics, Canada) at week 24, that is 16 weeks after STZ injection. Mice were anesthetized with isoflurane (1.5%–2.0%), and M-mode echocardiography was performed at the short-axis view of the left ventricle. Parameters including left ventricular end-diastolic diameter (LVEDD), left ventricular end-systolic diameter (LVESD), left ventricular ejection fraction (LVEF), and left ventricular fractional shortening (LVFS) were measured and analyzed using VevoLab software.

### 2.3 Biochemical analysis

Fasting blood glucose was measured using a glucose meter (Roche, Switzerland) monthly from week 0 to week 24. Serum levels of cardiac troponin I (cTnI), creatine kinase-MB (CK-MB), lactate dehydrogenase (LDH), and malondialdehyde (MDA) were detected using commercial ELISA kits (R&D Systems, United States) according to the manufacturer’s instructions.

### 2.4 Histological and immunofluorescence staining

Mouse hearts were harvested at week 24, fixed in 4% paraformaldehyde, embedded in paraffin, and cut into 5-μm sections. Hematoxylin-eosin (HE) staining was used to observe myocardial histopathological changes, and Masson’s trichrome staining was performed to evaluate myocardial fibrosis, with the collagen volume fraction quantified in ImageJ. Cardiomyocyte cross-sectional area was assessed by staining with FITC-conjugated wheat germ agglutinin (WGA, 10 μg/mL, Sigma-Aldrich, United States) for 1 h at room temperature, with at least 100 cardiomyocytes measured per animal in ImageJ.

For immunofluorescence staining, deparaffinized sections were subjected to antigen retrieval with citrate buffer (pH 6.0), blocked with 5% bovine serum albumin (BSA) for 1 h, and incubated with primary antibodies overnight at 4 °C. Primary antibodies included anti-PIK3R1 (1:500, Cell Signaling Technology, United States), anti-Nrf2 (1:500, Abcam, United Kingdom), anti-GPX4 (1:500, Abcam, United Kingdom), anti-ACSL4 (1:500, Abcam, United Kingdom), anti-TOMM20 (1:500, Cell Signaling Technology, United States), and anti-TUNEL (1:100, Roche, Switzerland). After washing with PBS, sections were incubated with fluorescent secondary antibodies (1:1000, Invitrogen, United States) for 1 h at room temperature. DAPI (1:1000, Invitrogen, United States) was used for nuclear staining. Images were captured using a fluorescence microscope (Olympus, Japan) under identical exposure settings for all groups, and positive cells were quantified using ImageJ software. Three animals per group were analysed, with five randomly selected non-overlapping fields per animal for histological and immunofluorescence sections; fields were selected by an investigator blinded to group allocation, and the mean of the fields was taken as the single value for that animal, so that the unit of analysis was the animal.

### 2.5 Metabolic and mitochondrial assays

For the intraperitoneal glucose tolerance test, mice were fasted for 12 h and given glucose 2 g/kg intraperitoneally, and tail blood glucose was measured at 0, 15, 30, 60 and 120 min with a glucose meter (Roche, Switzerland). Myocardial ATP content was determined in tissue lysates with an ATP assay kit (Beyotime, China) by the firefly luciferase method and normalised to protein concentration. Mitochondrial DNA copy number was determined by quantitative PCR of the mitochondrial gene mt-Nd1 relative to the nuclear gene Hk2 in total cardiac DNA. Reactive oxygen species were detected in fresh frozen sections with dihydroethidium (DHE, 10 μmol/L, Beyotime, China) for 30 min at 37 °C in the dark. Lipid peroxidation was assessed in fresh frozen sections with the ratiometric probe C11-BODIPY 581/591 (10 μmol/L, Thermo Fisher Scientific, United States) for 30 min at 37 °C, the reduced form being imaged in the red channel and the oxidised form in the green channel, and the result expressed as the proportion of reduced probe signal. Mitophagy was assessed by incubating sections with MitoTracker Red (200 nmol/L, Beyotime, China) followed by anti-LC3B (1:200, Cell Signaling Technology, United States) and a fluorescent secondary antibody. Images were acquired on a fluorescence microscope (Olympus, Japan) under identical exposure settings for all groups, and fluorescence intensity was quantified in ImageJ in five randomly selected fields per animal, the mean of the fields being taken as the value for that animal.

### 2.6 Quantitative real-time PCR

Total RNA was extracted from myocardial tissue with TRIzol reagent (Invitrogen, United States) and reverse transcribed with the PrimeScript RT reagent kit (TaKaRa, Japan). Quantitative PCR was performed with TB Green Premix Ex Taq II (TaKaRa, Japan) on a CFX96 system (Bio-Rad, United States), with 40 cycles of 95 °C for 5 s and 60 °C for 30 s. Relative expression was calculated by the 2^-ΔΔCt^ method with β-actin as the internal control. Primer sequences are given in Supplementary Table S1, and each sample was run in triplicate.

### 2.7 Transmission electron microscopy (TEM)

Myocardial tissue samples (1 mm^3^) were fixed in 2.5% glutaraldehyde overnight at 4 °C, post-fixed in 1% osmium tetroxide for 2 h, dehydrated in gradient ethanol, and embedded in epoxy resin. Ultrathin sections (70 nm) were stained with uranyl acetate and lead citrate, and observed under a transmission electron microscope (JEM-1400, JEOL, Japan) to assess mitochondrial morphology. Three animals per group were examined, with three randomly selected fields per animal chosen by an investigator blinded to group allocation, and the mean of the fields was taken as the value for that animal.

### 2.8 Iron content measurement

Myocardial tissue iron deposition was detected by Prussian blue staining. Paraffin sections were stained with Prussian blue solution (Sigma-Aldrich, United States) for 30 min, counterstained with nuclear fast red, and observed under a light microscope. The relative iron content was quantified using ImageJ software.

Intracellular iron content was measured using a ferrozine-based assay kit (Abcam, United Kingdom) according to the manufacturer’s instructions. Myocardial tissue was homogenized in lysis buffer, and the supernatant was mixed with ferrozine reagent. The absorbance was measured at 562 nm using a microplate reader (Thermo Fisher Scientific, United States), and iron content was calculated based on a standard curve.

### 2.9 Western blot analysis

Myocardial tissue was homogenized in RIPA lysis buffer (Beyotime, China) containing protease and phosphatase inhibitors (Roche, Switzerland). Protein concentration was determined using a BCA protein assay kit (Thermo Fisher Scientific, United States). Equal amounts of protein (50 μg) were separated by SDS-PAGE and transferred to PVDF membranes (Millipore, United States). Membranes were blocked with 5% non-fat milk for 1 h at room temperature, and incubated with primary antibodies overnight at 4 °C. Primary antibodies included anti-PIK3R1 (1:1000, Cell Signaling Technology, United States), anti-PI3K (1:1000, Cell Signaling Technology, United States), anti-p-Akt (1:1000, Cell Signaling Technology, United States), anti-Akt (1:1000, Cell Signaling Technology, United States), anti-Nrf2 (1:1000, Abcam, United Kingdom), anti-GPX4 (1:1000, Abcam, United Kingdom), anti-ACSL4 (1:1000, Abcam, United Kingdom), anti-SLC7A11 (1:1000, Abcam, United Kingdom), anti-Drp1 (1:1000, Cell Signaling Technology, United States), anti-Mfn2 (1:1000, Cell Signaling Technology, United States), anti-OPA1 (1:1000, Cell Signaling Technology, United States), anti-Bax (1:1000, Cell Signaling Technology, United States), anti-Bcl-2 (1:1000, Cell Signaling Technology, United States), and anti-GAPDH (1:5000, Santa Cruz Biotechnology, United States). After washing with TBST, membranes were incubated with horseradish peroxidase (HRP)-conjugated secondary antibodies (1:5000, Santa Cruz Biotechnology, United States) for 1 h at room temperature. Protein bands were visualized using an enhanced chemiluminescence (ECL) detection kit (Millipore, United States) and quantified using ImageJ software. Phosphorylated proteins were normalised to the corresponding total protein on the same membrane, nuclear Nrf2 was normalised to Histone H3, and all other proteins were normalised to GAPDH or β-actin as indicated in the figures.

### 2.10 Bioinformatic analysis

Myocardial transcriptome data from mouse models of diabetic cardiomyopathy were retrieved from the Gene Expression Omnibus (GEO). Differentially expressed genes (DEGs) between DCM and control myocardium were identified with the limma package in R (version 4.2.2), using thresholds of P < 0.05 and |log2 fold change| > 0.5, and were displayed as a volcano plot. Upregulated DEGs were intersected with the mouse MitoCarta 3.0 inventory of mitochondrial genes and with the ferroptosis-driver gene set of FerrDb V2, and the intersection was displayed as a Venn diagram. Expression of the resulting candidate genes was examined in three independent GEO datasets (GSE161052, GSE161931 and GSE215979) and compared between groups with the Wilcoxon rank-sum test. KEGG pathway enrichment analysis of the upregulated and downregulated DEGs was performed with the clusterProfiler package, and pathways with P < 0.05 were considered significantly enriched.

### 2.11 Statistical analysis

All data were expressed as mean ± standard deviation (SD). Statistical analysis was performed using SPSS 22.0 software (IBM, United States) and GraphPad Prism 8.0 software (GraphPad Software, United States). Normality was assessed by the Shapiro-Wilk test and homogeneity of variance by Levene’s test; all datasets satisfied both assumptions, and no data transformation was applied. Comparisons between two groups used the unpaired two-tailed Student’s t-test. Comparisons among three or more groups used one-way analysis of variance (ANOVA) followed by Tukey’s *post hoc* test, and the cohort comprising two genotypes and three treatments was analysed by two-way ANOVA with Tukey’s *post hoc* test. No outliers were excluded and all acquired data points are shown in the figures. Mice were assigned to groups by a computer-generated random sequence, and echocardiographic measurement, histological scoring and image quantification were performed by investigators blinded to group allocation. Group size was based on established practice in comparable models of diabetic cardiomyopathy rather than on an *a priori* power calculation. P < 0.05 was considered statistically significant.

## 3 Results

### 3.1 PIK3R1 is identified as a key upregulated gene in diabetic cardiomyopathy via omics analysis

Bioinformatics analysis was performed to screen for differentially expressed genes (DEGs) associated with diabetic cardiomyopathy (DCM), and the volcano plot of DEGs in DCM samples versus normal cardiac tissues revealed that PIK3R1 was significantly upregulated (*P* < 0.05, |log2 fold change| > 0.5) (Figure 1A). Intersection analysis of upregulated DEGs, MitoCarta 3.0 database, and FerrDb ferroptosis-promoting gene set via Venn diagram further confirmed the correlation of PIK3R1 with mitochondrial function and ferroptosis (Figure 1B). Expression of the four intersecting genes (Acsl4, Aifm2, Decr1 and Pik3r1) was examined in three independent DCM datasets (GSE161052, GSE161931 and GSE215979), and Pik3r1 was consistently higher in the DCM groups than in the normal groups (Figure 1C). KEGG enrichment analysis of the differentially expressed genes showed that the PI3K-Akt signaling pathway was the most significantly enriched upregulated pathway (Figure 1D) Collectively, these omics data identified PIK3R1 as a core differentially expressed gene closely related to DCM, mitochondrial homeostasis, and ferroptosis.

**FIGURE 1:**
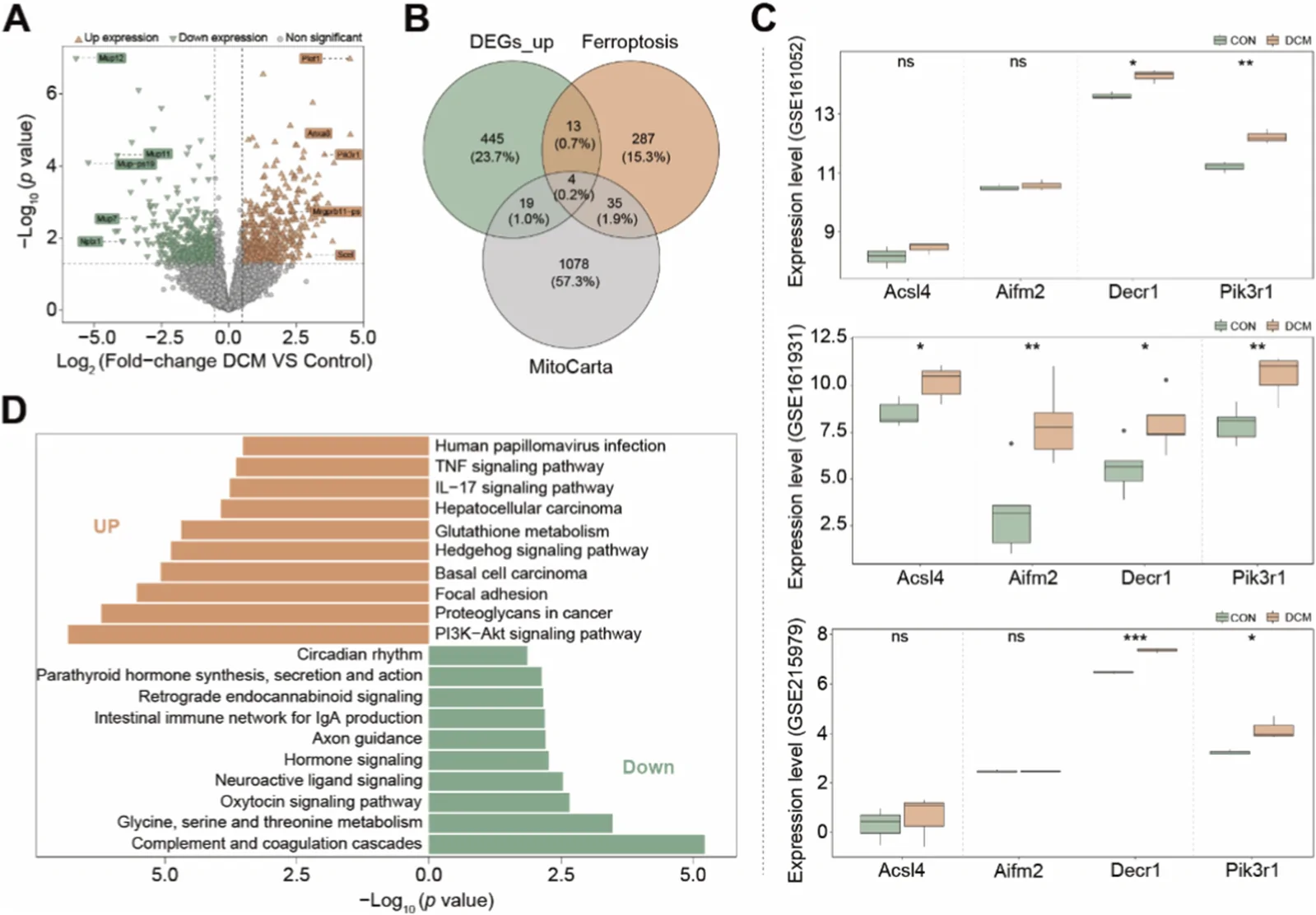
Omics analysis identifies PIK3R1 as a significantly differentially expressed gene in diabetic cardiomyopathy. **(A)** Volcano plot showing the differential expression profile of the DCM dataset; PIK3R1 is prominently displayed as a significantly upregulated gene. **(B)** Venn diagram illustrating the intersection analysis of upregulated differentially expressed genes (Up-DEGs), MitoCarta 3.0, and ferroptosis-promoting gene sets from FerrDb. **(C)** Validation of PIK3R1 expression in normal and DCM groups across independent external datasets. **(D)** KEGG pathway enrichment analysis of the upregulated and downregulated differentially expressed genes.

### 3.2 PIK3R1 is overexpressed in the myocardium of a successfully established DCM mouse model, and PIK3R1 upregulation is associated with DCM progression *in vivo*

A DCM mouse model was constructed by high-fat diet (HFD) feeding combined with low-dose streptozotocin (STZ) injection, and the experimental workflow including modeling time nodes, interventions, grouping, phenotypic identification, and sampling was schematized (Figure 2A). Echocardiographic assessment at week 24 showed that compared with the control (CON) group, DCM mice exhibited impaired cardiac systolic function, with significantly reduced LVEF and LVFS (Figure 2B). Detection of fasting blood glucose and intraperitoneal glucose tolerance test (IPGTT) showed that DCM mice had significantly elevated fasting blood glucose, and their glucose regulation ability was impaired with a peak blood glucose at 30 min and sustained high blood glucose within 120 min after glucose load (Figure 2C). Hematoxylin-eosin (HE) staining, Wheat Germ Agglutinin (WGA) staining, and Masson’s trichrome staining of myocardial tissues revealed that DCM mice presented obvious myocardial remodeling, including increased cardiomyocyte cross-sectional area (myocardial hypertrophy) and enhanced myocardial collagen deposition (myocardial fibrosis) (Figure 2D). qRT-PCR detection at the transcriptional level confirmed that PIK3R1 mRNA expression in the myocardium of DCM mice was significantly higher than that in the CON group (Figure 2E). Western blot analysis further verified the significant upregulation of PIK3R1 protein expression in DCM myocardial tissues (Figure 2F). Immunofluorescence double staining with anti-PIK3R1 and cardiomyocyte-specific anti-cTnT antibodies showed that PIK3R1 was specifically overexpressed in cardiomyocytes of DCM mice, and quantitative analysis of fluorescence intensity confirmed the significant increase in PIK3R1 expression (Figure 2G). All the above results indicated the successful establishment of the DCM mouse model with PIK3R1 myocardial overexpression, and PIK3R1 is associated with the occurrence and development of DCM.

**FIGURE 2:**
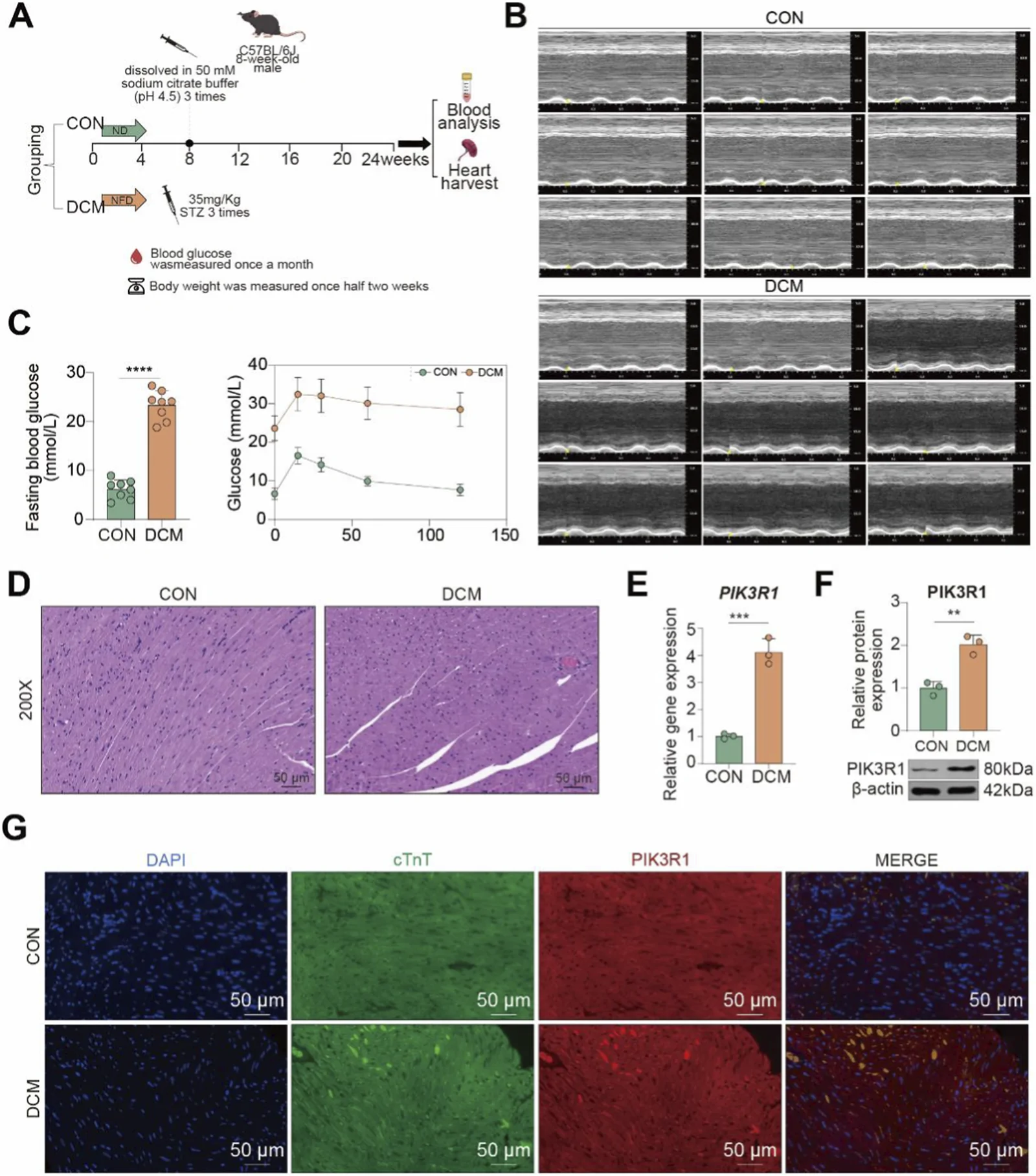
*In vivo* characterization of the effect of PIK3R1 on the progression of diabetic cardiomyopathy. **(A)** Schematic diagram of DCM mouse model establishment, showing modeling time nodes, interventions, experimental grouping, phenotypic identification methods, and tissue collection time points. **(B)** Establishment and validation of the DCM mouse model–echocardiographic assessment of cardiac function. **(C)** Establishment and validation of the DCM mouse model–detection of fasting blood glucose and glucose tolerance in mice. **(D)** Establishment and validation of the DCM mouse model–Hematoxylin-eosin (HE) staining of myocardial tissues. **(E)** Validation of PIK3R1 expression levels–qRT-PCR analysis of PIK3R1 transcriptional changes in the DCM group. **(F)** Validation of PIK3R1 expression levels–Western blot analysis of PIK3R1 protein changes in the DCM group. **(G)** Validation of PIK3R1 expression levels–Immunofluorescence analysis of PIK3R1 expression and distribution in cardiomyocytes of the DCM group.

### 3.3 Inhibition of cardiomyocyte ferroptosis alleviates DCM progression *in vivo*

To explore the role of ferroptosis in DCM, Ferrostatin-1 (Fer-1), a specific ferroptosis inhibitor, was used to intervene in DCM mice, and the experimental grouping and intervention strategy were schematized (Figure 3A). Echocardiographic results showed that compared with the CON group, DCM mice had significantly decreased LVEF and LVFS; while Fer-1 treatment significantly improved cardiac systolic function of DCM mice, with increased LVEF and LVFS (Figure 3B, color scheme: CON#9ebfe5, DCM#f1a09d, Fer-1#c0e2d2). HE staining results showed that Fer-1 treatment alleviated myocardial remodeling in DCM mice, including reduced myocardial pathological damage, alleviated myocardial fibrosis, and improved myocardial hypertrophy (Figure 3C). Biochemical detection of serum myocardial function factors revealed that the levels of creatine kinase (CK), creatine kinase-MB (CK-MB), and lactate dehydrogenase (LDH) in DCM mice were significantly higher than those in the CON group, and Fer-1 treatment significantly reduced the levels of these injury factors (Figure 3D). TUNEL assay showed that myocardial cell apoptosis was significantly increased in DCM mice, while Fer-1 intervention significantly reduced the number of TUNEL-positive apoptotic cells (Figure 3E). Western blot detection of apoptosis-related proteins showed that compared with the CON group, DCM mice had downregulated Bcl-2 and upregulated Bax and cleaved caspase3; Fer-1 treatment reversed the expression of these proteins, with increased Bcl-2 and decreased Bax and cleaved caspase3 (Figure 3F). These results suggested that ferroptosis is involved in DCM pathogenesis, and inhibition of ferroptosis can alleviate myocardial injury, reduce cardiomyocyte apoptosis, and improve cardiac function in DCM mice.

**FIGURE 3:**
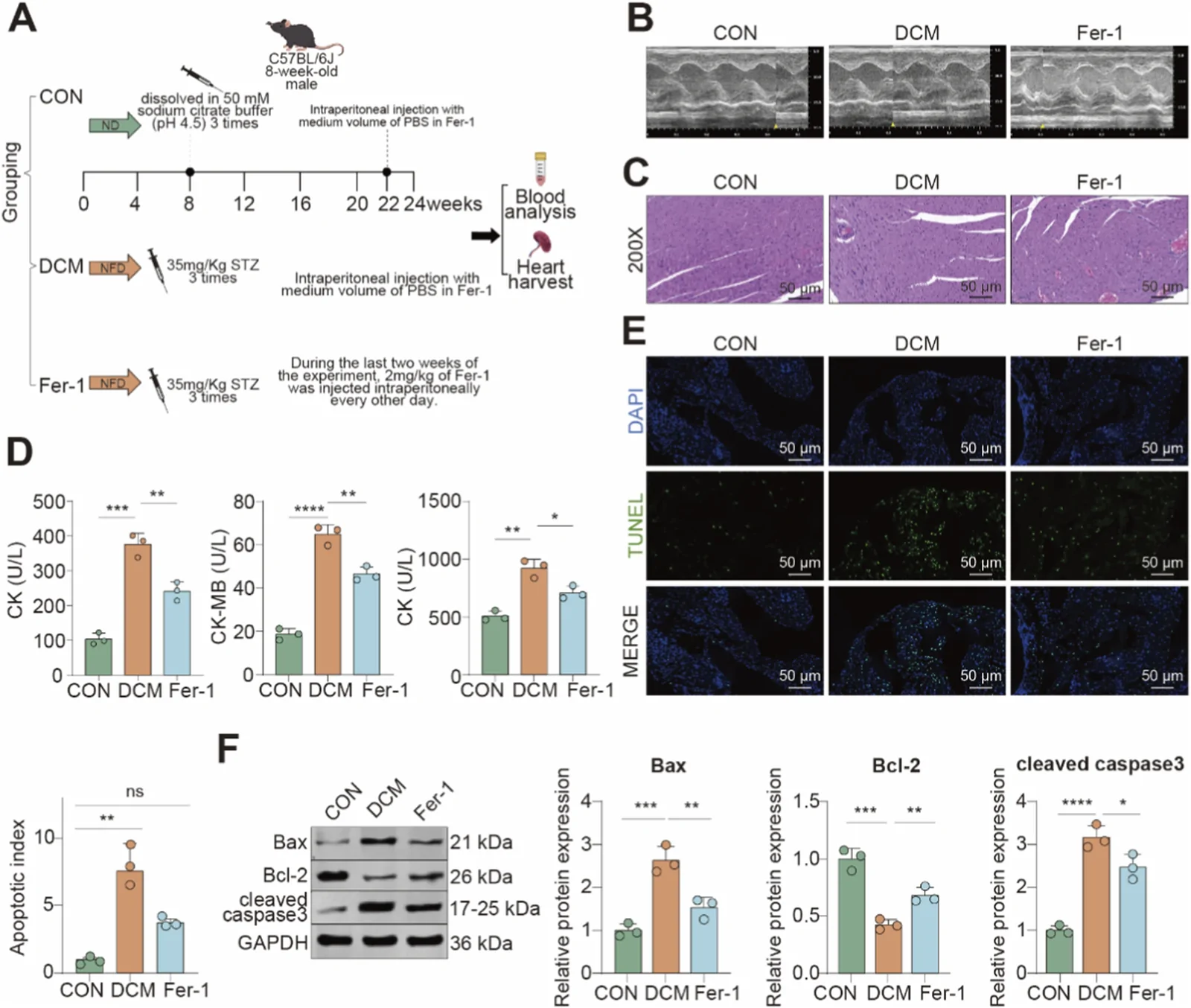
*In vivo* investigation of the effect of cardiomyocyte ferroptosis on the progression of diabetic cardiomyopathy. **(A)** Schematic diagram of experimental grouping of DCM mice, showing modeling time nodes, interventions, experimental grouping, phenotypic identification methods, and tissue collection time points. **(B)** Cardiac function of DCM mice–echocardiographic assessment. **(C)** Cardiac function of DCM mice–HE staining of myocardial tissues. **(D)** Cardiac function indices of DCM mice–biochemical detection of myocardial function factors in serum. **(E)** Myocardial apoptosis in DCM mice–TUNEL assay for the detection of cardiomyocyte apoptosis. **(F)** Myocardial apoptosis in DCM mice–Western blot analysis of apoptosis-related proteins.

### 3.4 Improvement of mitochondrial homeostasis alleviates DCM progression *in vivo*

Mdivi-1, a specific mitochondrial fission inhibitor, was used to intervene in DCM mice to explore the role of mitochondrial homeostasis in DCM, and the experimental grouping and intervention process were schematized (Figure 4A). Echocardiographic assessment showed that compared with the CON group, DCM mice had significantly impaired cardiac function with decreased LVEF and LVFS; Mdivi-1 treatment significantly improved the cardiac systolic function of DCM mice (Figure 4B). HE staining, Masson’s trichrome staining, and WGA staining showed that DCM mice had severe myocardial remodeling, and Mdivi-1 treatment significantly alleviated myocardial pathological damage, reduced myocardial fibrosis, and improved myocardial hypertrophy (Figure 4C). Biochemical detection of serum showed that Mdivi-1 treatment significantly reduced the levels of CK, CK-MB, and LDH in DCM mice, indicating alleviated myocardial injury (Figure 4D). TUNEL assay revealed that Mdivi-1 intervention significantly reduced the number of apoptotic cardiomyocytes in DCM mice (Figure 4E). Western blot analysis of apoptosis-related proteins showed that Mdivi-1 treatment upregulated Bcl-2 and downregulated Bax and cleaved caspase3 in DCM myocardial tissues, consistent with the results of the TUNEL assay (Figure 4F). These results indicated that mitochondrial fission disorder and homeostasis imbalance are important factors in DCM progression, and inhibition of mitochondrial fission can alleviate myocardial injury and apoptosis in DCM mice.

**FIGURE 4:**
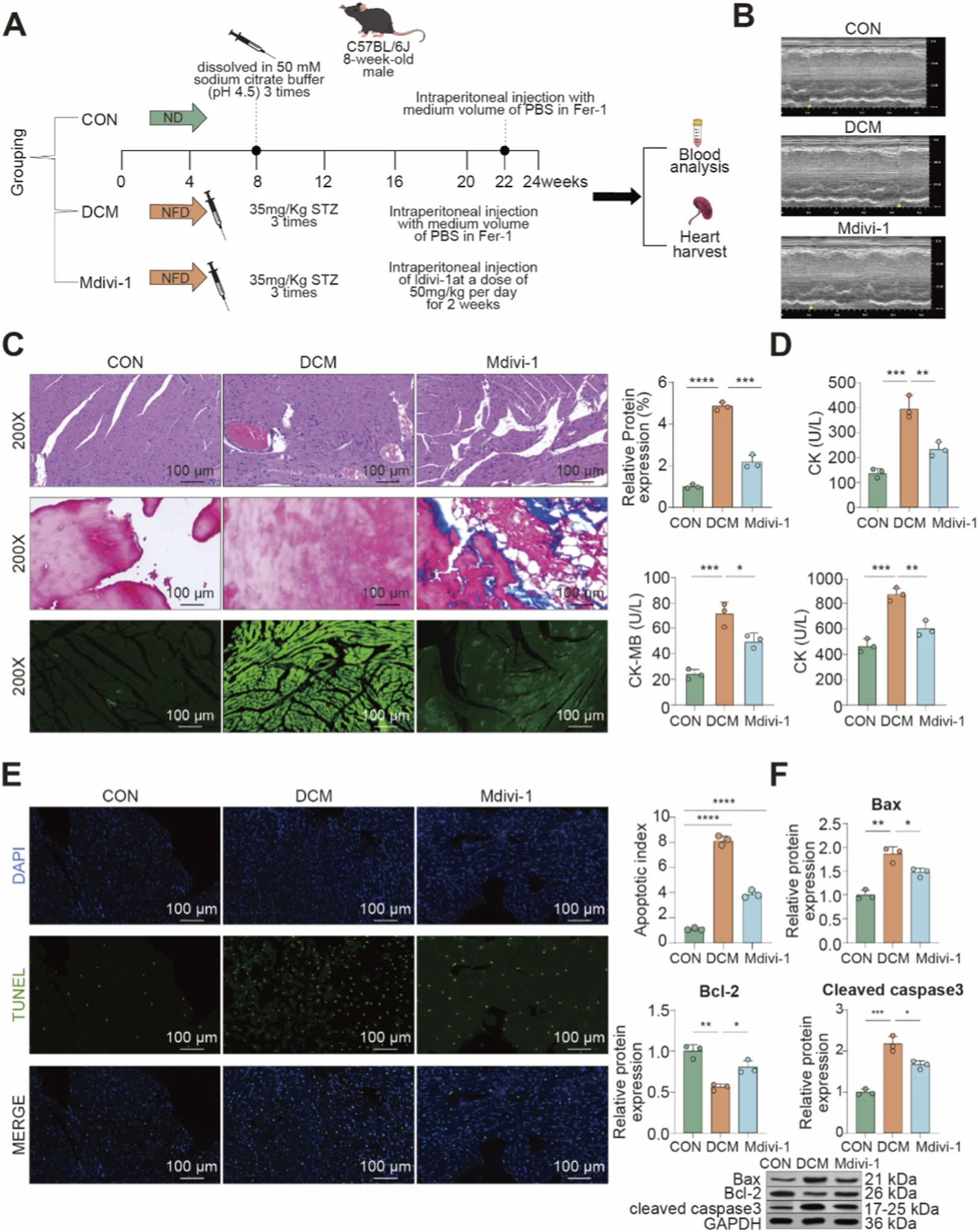
*In vivo* exploration of the effect of mitochondrial homeostasis imbalance on the progression of diabetic cardiomyopathy. **(A)** Schematic diagram of experimental grouping of DCM mice, showing modeling time nodes, interventions, experimental grouping, phenotypic identification methods, and tissue collection time points. **(B)** Cardiac function of DCM mice–echocardiographic assessment. **(C)** Cardiac function of DCM mice–pathological staining (HE staining, Masson’s trichrome staining, and WGA staining) of myocardial tissues. **(D)** Cardiac function indices of DCM mice–biochemical detection of myocardial function factors in serum. **(E)** Myocardial apoptosis in DCM mice–TUNEL assay for the detection of cardiomyocyte apoptosis. **(F)** Myocardial apoptosis in DCM mice–Western blot analysis of apoptosis-related proteins.

### 3.5 Abnormal mitochondrial homeostasis is associated with cardiomyocyte ferroptosis in DCM mice *in vivo*

Transmission electron microscopy (TEM) was used to observe the ultrastructure of myocardial mitochondria, and the results showed that compared with the CON group, mitochondria in DCM mice presented atrophy, increased membrane density, cristae disruption, and abnormal morphology; Mdivi-1 treatment significantly improved mitochondrial ultrastructural damage in DCM mice (Figure 5A). Prussian blue staining of myocardial tissues showed that DCM mice had obvious myocardial iron deposition, while Mdivi-1 treatment significantly reduced iron deposition (Figure 5B). Biochemical kit detection further confirmed that the Fe^2+^ content in the myocardium of DCM mice was significantly higher than that in the CON group, and Mdivi-1 intervention significantly decreased the Fe^2+^ level (Figure 5C). Detection of lipid peroxide levels showed that compared with the CON group, DCM mice had increased malondialdehyde (MDA) and decreased glutathione (GSH) and GSH/GSSG ratio in myocardial tissues; Mdivi-1 treatment reversed these changes, with decreased MDA and increased GSH and GSH/GSSG ratio (Figure 5D). C11-BODIPY fluorescent probe staining showed that the CON group mainly presented red fluorescence (non-lipid peroxidation state), DCM mice had significantly reduced red fluorescence and increased green fluorescence (severe lipid peroxidation), and Mdivi-1 treatment increased red fluorescence and decreased green fluorescence in DCM mice (Figure 5E). Western blot detection of ferroptosis-related proteins showed that DCM mice had downregulated GPX4 and SLC7A11 and upregulated ACSL4 in myocardial tissues; Mdivi-1 treatment upregulated GPX4 and SLC7A11 and downregulated ACSL4 (Figure 5F). These results demonstrated that abnormal mitochondrial homeostasis in DCM induces cardiomyocyte ferroptosis by promoting iron deposition and lipid peroxidation, and improvement of mitochondrial homeostasis can inhibit cardiomyocyte ferroptosis.

**FIGURE 5:**
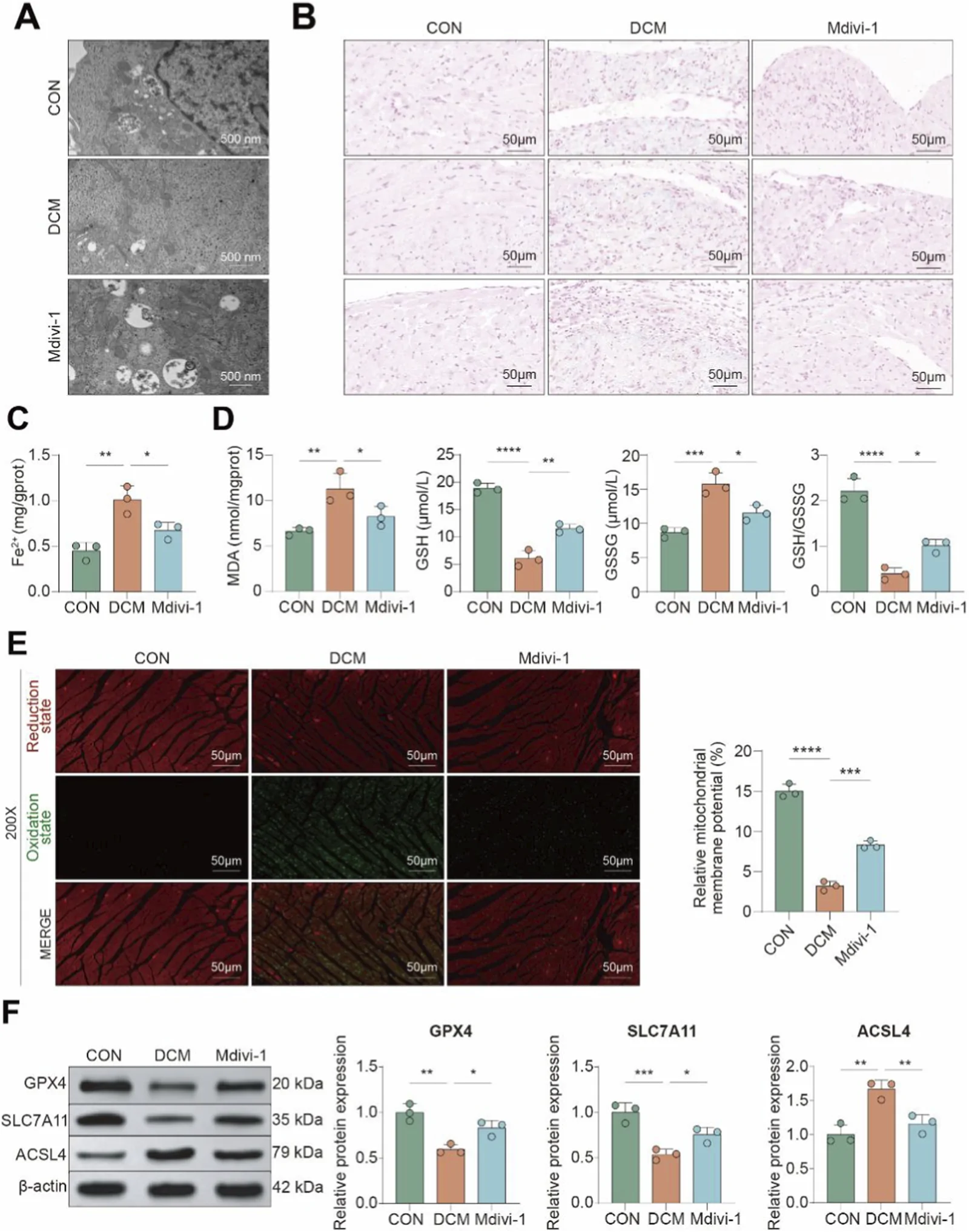
*In vivo* elucidation of the mechanism by which abnormal mitochondrial homeostasis induces cardiomyocyte ferroptosis in diabetic cardiomyopathy. **(A)** Transmission electron microscopy (TEM) observation of the ultrastructure of mitochondrial in cardiomyocytes. **(B)** Myocardial iron deposition in DCM mice–Prussian blue staining of myocardial tissues. **(C)** Myocardial iron deposition in DCM mice–quantification of Fe^2+^ content in myocardial tissues using a biochemical kit. **(D)** Myocardial lipid peroxide levels in DCM mice–detection of GSH, GSH/GSSG ratio and MDA levels using biochemical kits. **(E)** Myocardial lipid peroxide levels in DCM mice, assessed with the ratiometric C11-BODIPY 581/591 probe; the reduced form is shown in red and the oxidised form in green, and the quantification gives the reduced signal as a percentage of the total. **(F)** Cardiomyocyte ferroptosis in DCM mice–Western blot analysis of ferroptosis-related proteins.

### 3.6 PIK3R1 deficiency is associated with improved mitochondrial homeostasis and increased PI3K/Akt/Nrf2 signaling in DCM mice *in vivo*

Wild-type (WT) mice and PIK3R1 knockout (PIK3R1KO) mice were used to construct DCM models, and the PI3K/Akt agonist 740 Y-P was used for intervention; the experimental grouping was schematized (Figure 6A). Western blot detection of PI3K/Akt/Nrf2 signaling pathway-related proteins showed that compared with WT-CON, WT-DCM mice had decreased phosphorylation levels of PI3K and Akt (p-PI3K, p-Akt), and reduced nuclear Nrf2 and HO-1 protein (t-PI3K and t-Akt remained unchanged); 740 Y-P treatment increased the above proteins in WT-DCM mice. PIK3R1KO mice showed the same expression trend of these proteins as WT mice, and the levels of p-PI3K, p-Akt, nuclear Nrf2 and HO-1 in PIK3R1KO mice were significantly higher than those in WT mice under the same treatment (PIK3R1KO-CON ≈ WT-CON, PIK3R1KO-DCM > WT-DCM, PIK3R1KO-740 Y-P > WT-740 Y-P) (Figure 6B; Nuclear Nrf2 internal reference: Histone H3, others: β-actin). DHE fluorescent probe detection of reactive oxygen species (ROS) showed that WT-DCM mice had significantly increased myocardial ROS production, and 740 Y-P treatment reduced ROS levels; PIK3R1KO mice had the same ROS change trend as WT mice, and the ROS level in PIK3R1KO mice was lower than that in WT mice under the same treatment (PIK3R1KO-CON ≈ WT-CON) (Figure 6C). Detection of mitochondrial ATP production showed that WT-DCM mice had significantly decreased myocardial ATP content, and 740 Y-P treatment increased ATP levels; PIK3R1KO mice had the same ATP change trend as WT mice, and the ATP content in PIK3R1KO mice was higher than that in WT mice under the same treatment (Figure 6D). Detection of mitochondrial DNA (mtDNA) copy number showed that the change trend of mtDNA in each group was consistent with that of ATP (Figure 6E). Mito Tracker and LC3B double immunofluorescence staining showed that mitochondrial autophagy was impaired in WT-DCM mice, and 740 Y-P treatment improved mitochondrial autophagy; PIK3R1KO mice had the same mitochondrial autophagy change trend as WT mice, and mitochondrial autophagy in PIK3R1KO mice was more active than that in WT mice under the same treatment (Figure 6F). Since p85α encoded by PIK3R1 constrains PI3K activity, the significantly higher p-PI3K and p-Akt levels in PIK3R1KO-DCM myocardium than in WT-DCM myocardium under identical conditions, in the absence of any difference between the two control genotypes, are consistent with effective loss of cardiomyocyte PIK3R1. These results indicated that PIK3R1 deficiency is associated with increased PI3K/Akt/Nrf2 signaling, and that PIK3R1 knockout or pharmacological PI3K/Akt activation is accompanied by improved mitochondrial function and homeostasis in DCM mice.

**FIGURE 6:**
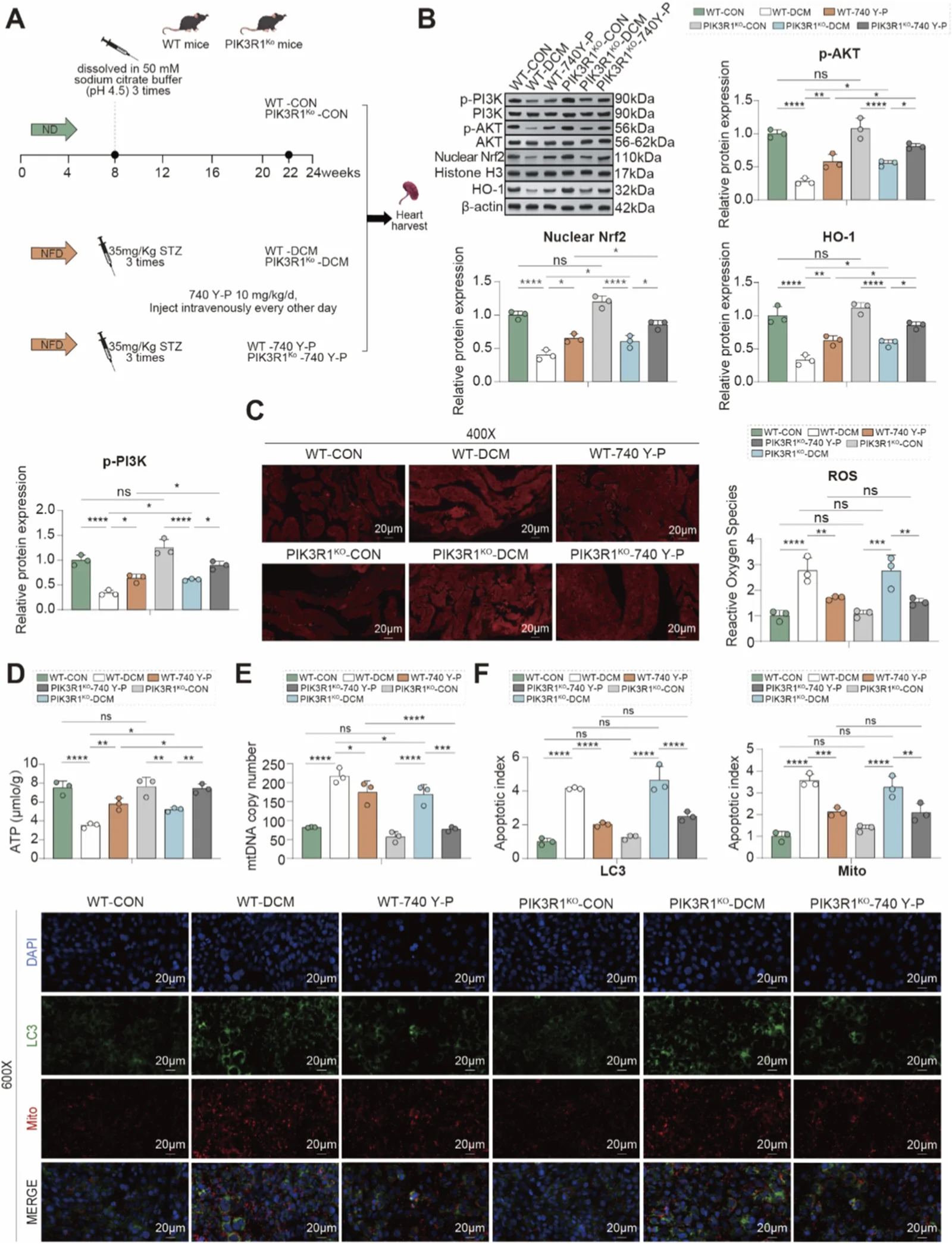
*In vivo* identification of the mechanism by which PIK3R1 regulates mitochondrial homeostasis via the PI3K/Akt/Nrf2 signaling pathway in diabetic cardiomyopathy. **(A)** Schematic diagram of experimental grouping, showing modeling time nodes, interventions, experimental grouping, phenotypic identification methods, and tissue collection time points. **(B)** The PI3K/Akt/Nrf2 signaling pathway in DCM mice–Western blot analysis of pathway-related proteins in myocardial tissues. **(C)** Mitochondrial injury in DCM mice–fluorescence microscopy observation of reactive oxygen species (ROS) production. **(D)** Mitochondrial injury in DCM mice–measurement of mitochondrial ATP production in myocardial tissues. **(E)** Mitochondrial injury in DCM mice–quantification of mitochondrial DNA (mtDNA) copy number in myocardial tissues. **(F)** Mitochondrial homeostasis in DCM mice–observation of mitophagy via Mito Tracker and LC3B double immunofluorescence staining.

### 3.7 PIK3R1 deficiency is associated with restored mitochondrial dynamic homeostasis in DCM mice *in vivo*

Immunofluorescence detection of Drp1, a key factor of mitochondrial fission, showed that in both WT and PIK3R1KO mice, DCM models had significantly increased myocardial Drp1 expression compared with the CON group, and 740 Y-P treatment reduced Drp1 expression; under the same treatment, Drp1 expression in PIK3R1KO mice was lower than that in WT mice (PIK3R1KO-CON ≈ WT-CON) (Figure 7A). Western blot detection of mitochondrial dynamic homeostasis-related factors and quantitative analysis via heat map showed that in both WT and PIK3R1KO mice, compared with the CON group, DCM mice had downregulated LC3-II/I, Mfn2, and Opa1 and upregulated p62, Drp1, and Fis1; 740 Y-P treatment reversed the expression of these proteins. Under the same treatment, PIK3R1KO mice had higher LC3-II/I, Mfn2, and Opa1 and lower p62, Drp1, and Fis1 than WT mice (Figure 7B). These results confirmed that PIK3R1 regulates mitochondrial dynamic homeostasis in DCM mice by modulating the expression of mitochondrial fission-fusion and autophagy-related factors, and PIK3R1 knockout can restore the balance of mitochondrial dynamics.

**FIGURE 7:**
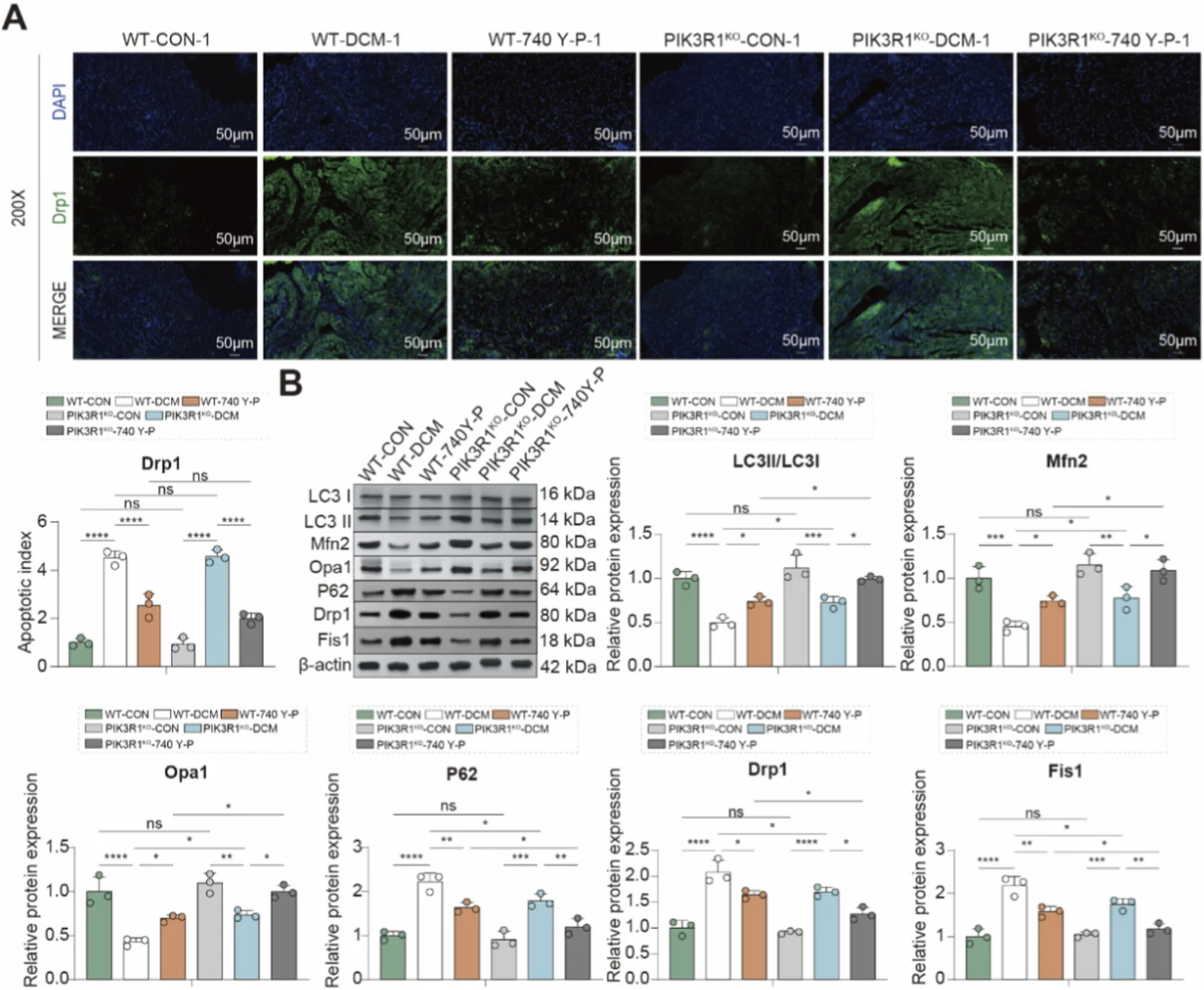
*In vivo* further confirmation that mitochondrial homeostasis in cardiomyocytes is regulated by PIK3R1 in diabetic cardiomyopathy. **(A)** Mitochondrial homeostasis in DCM mice–immunofluorescence detection of Drp1, a key factor of mitochondrial fission and fusion. **(B)** Mitochondrial homeostasis in DCM mice–Western blot analysis of key factors involved in mitochondrial dynamic homeostasis.

### 3.8 PIK3R1 deficiency is associated with reduced cardiomyocyte ferroptosis and increased PI3K/Akt/Nrf2 signaling in DCM mice *in vivo*

TEM observation of myocardial mitochondrial ultrastructure showed that in both WT and PIK3R1KO mice, DCM models had abnormal mitochondrial morphology (atrophy, increased membrane density) compared with the CON group, and 740 Y-P treatment improved mitochondrial ultrastructure; under the same treatment, mitochondrial damage in PIK3R1KO mice was milder than that in WT mice (PIK3R1KO-CON ≈ WT-CON) (Figure 8A). Prussian blue staining showed that in both WT and PIK3R1KO mice, DCM models had significantly increased myocardial iron deposition compared with the CON group, and 740 Y-P treatment reduced iron deposition; under the same treatment, iron deposition in PIK3R1KO mice was less than that in WT mice (Figure 8B). Biochemical kit detection confirmed that the change trend of myocardial Fe^2+^ content in each group was consistent with the results of Prussian blue staining (Figure 8C). Detection of lipid peroxide levels (color scheme: WT-CON#9ebfe5, WT-DCM#f1a09d, WT-740 Y-P#c0e2d2, PIK3R1KO-CON#f9c77e) showed that in both WT and PIK3R1KO mice, DCM models had increased myocardial MDA and decreased GSH and GSH/GSSG ratio compared with the CON group, and 740 Y-P treatment reversed these changes; under the same treatment, PIK3R1KO mice had lower MDA and higher GSH and GSH/GSSG ratio than WT mice (Figure 8D). C11-BODIPY fluorescent probe staining showed that WT-CON and PIK3R1KO-CON groups mainly presented red fluorescence; DCM groups had significantly reduced red fluorescence and increased green fluorescence, and 740 Y-P treatment increased red fluorescence and decreased green fluorescence; lipid peroxidation in PIK3R1KO-DCM mice was milder than that in WT-DCM mice, and the same trend was observed in the 740 Y-P intervention groups (Figure 8E). Western blot detection of ferroptosis-related proteins showed that in both WT and PIK3R1KO mice, DCM models had downregulated GPX4 and SLC7A11 and upregulated ACSL4 compared with the CON group, and 740 Y-P treatment reversed the expression of these proteins; under the same treatment, the change degree of these proteins in PIK3R1KO mice was milder than that in WT mice (PIK3R1KO-CON ≈ WT-CON) (Figure 8F). These results indicated that PIK3R1 knockout, or pharmacological activation of PI3K/Akt signalling, is accompanied by reduced myocardial iron deposition and lipid peroxidation in DCM mice.

**FIGURE 8:**
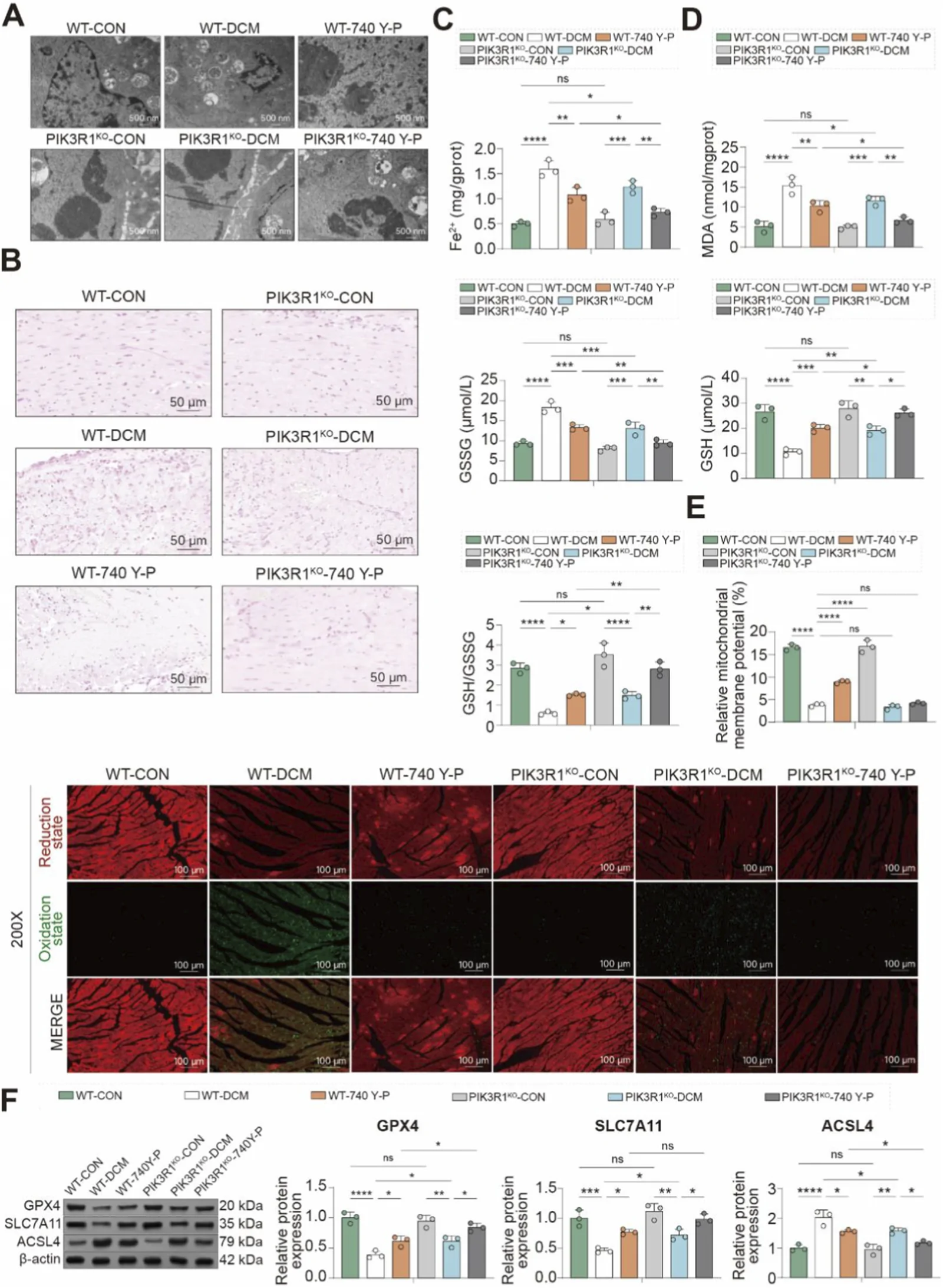
*In vivo* clarification of the mechanism by which PIK3R1 regulates cardiomyocyte ferroptosis via the PI3K/Akt/Nrf2 signaling pathway in diabetic cardiomyopathy. **(A)** Transmission electron microscopy (TEM) observation of the ultrastructure of mitochondrial in cardiomyocytes. **(B)** Myocardial iron deposition in DCM mice–Prussian blue staining of myocardial tissues. **(C)** Myocardial iron deposition in DCM mice–quantification of Fe^2+^ content in myocardial tissues using a biochemical kit. **(D)** Myocardial lipid peroxide levels in DCM mice–detection of GSH, GSH/GSSG ratio and MDA levels using biochemical kits. **(E)** Myocardial lipid peroxide levels in DCM mice, assessed with the ratiometric C11-BODIPY 581/591 probe; the reduced form is shown in red and the oxidised form in green, and the quantification gives the reduced signal as a percentage of the total. **(F)** Cardiomyocyte ferroptosis in DCM mice–Western blot analysis of ferroptosis-related proteins.

### 3.9 PIK3R1 deficiency attenuates DCM progression *in vivo*

WT mice and PIK3R1KO mice were used to construct DCM models to verify the regulatory effect of PIK3R1 on DCM progression, and the experimental grouping was schematized (Figure 9A). Echocardiographic results showed that there was no significant difference in LVEF and LVFS between WT-CON and PIK3R1KO-CON groups; compared with the CON group, DCM mice had significantly decreased cardiac function, and the cardiac function of PIK3R1KO-DCM mice was significantly better than that of WT-DCM mice (cardiac function trend: WT-CON ≈ PIK3R1KO-CON > PIK3R1KO-DCM > WT-DCM) (Figure 9B). HE staining, Masson’s trichrome staining, and WGA staining showed that there was no significant difference in myocardial morphology between WT-CON and PIK3R1KO-CON groups; DCM mice had obvious myocardial remodeling, and the myocardial remodeling degree of PIK3R1KO-DCM mice was significantly milder than that of WT-DCM mice (pathological severity trend: WT-CON ≈ PIK3R1KO-CON < PIK3R1KO-DCM < WT-DCM) (Figure 9C). Biochemical detection of serum showed that there was no significant difference in the levels of CK, CK-MB, and LDH between WT-CON and PIK3R1KO-CON groups; DCM mice had significantly increased levels of these injury factors, and the levels in PIK3R1KO-DCM mice were significantly lower than those in WT-DCM mice (factor level trend: WT-CON ≈ PIK3R1KO-CON < PIK3R1KO-DCM < WT-DCM) (Figure 9D). TUNEL assay showed that there was no significant difference in the number of apoptotic cardiomyocytes between WT-CON and PIK3R1KO-CON groups; DCM mice had significantly increased apoptosis, and the apoptosis level of PIK3R1KO-DCM mice was significantly lower than that of WT-DCM mice (apoptosis trend: WT-CON ≈ PIK3R1KO-CON < PIK3R1KO-DCM < WT-DCM) (Figure 9E). Western blot detection of apoptosis-related proteins showed that the change trend of Bax and cleaved caspase3 was consistent with the apoptosis trend, and Bcl-2 showed the opposite trend (Figure 9F). These results confirmed that PIK3R1 promotes DCM progression by inducing myocardial injury, cardiomyocyte apoptosis, abnormal mitochondrial homeostasis, and ferroptosis, and PIK3R1 knockout has a cardioprotective effect on DCM mice.

**FIGURE 9:**
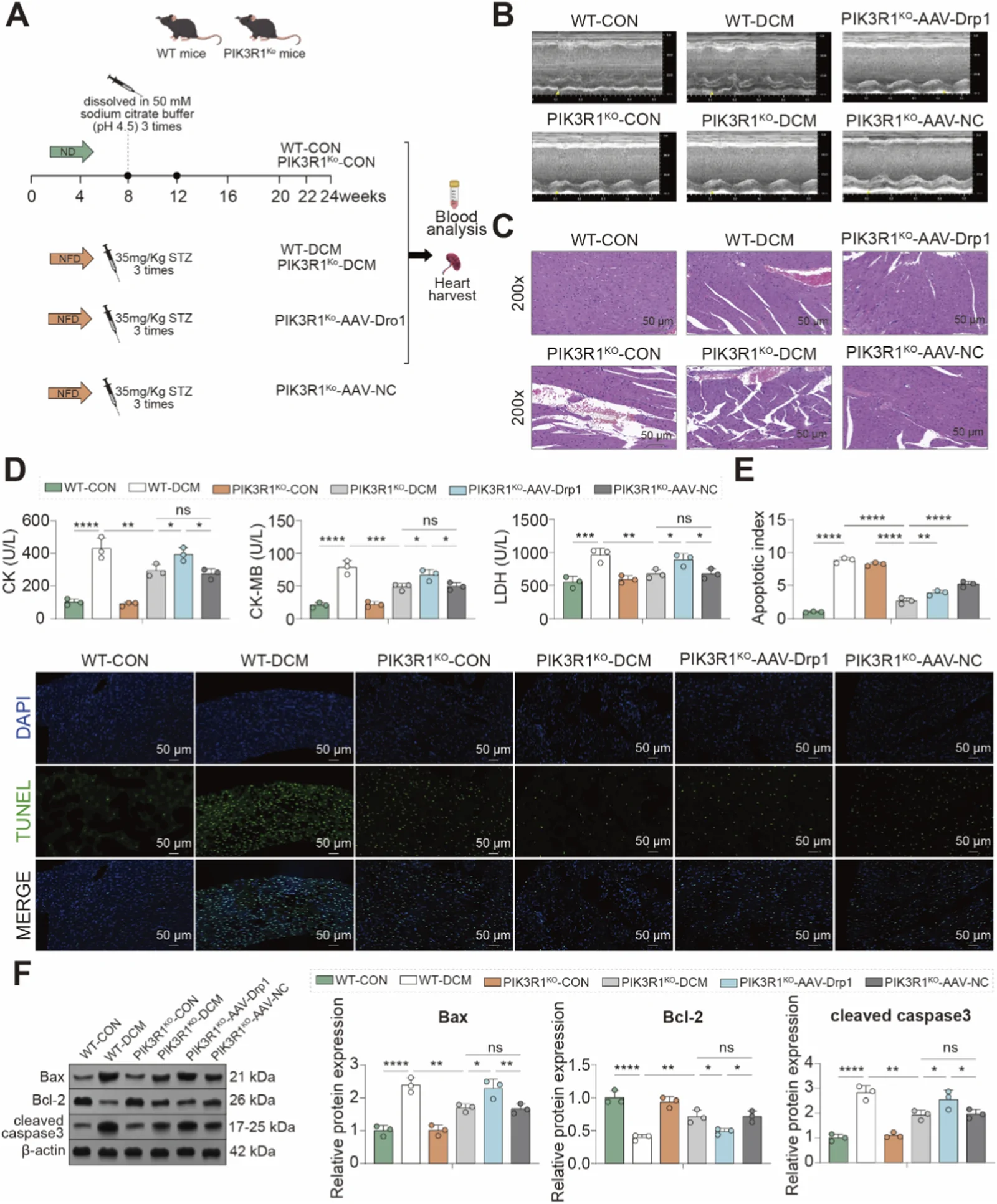
*In vivo* confirmation that PIK3R1 promotes diabetic cardiomyopathy progression by inducing abnormal mitochondrial homeostasis and subsequent cardiomyocyte ferroptosis. **(A)** Schematic diagram of experimental grouping, showing modeling time nodes, interventions, experimental grouping, phenotypic identification methods, and tissue collection time points. **(B)** Cardiac function of DCM mice–echocardiographic assessment. **(C)** Cardiac function of DCM mice–HE staining of myocardial tissues. **(D)** Cardiac function indices of DCM mice–biochemical detection of myocardial function factors in serum. **(E)** Myocardial apoptosis in DCM mice–TUNEL assay for the detection of cardiomyocyte apoptosis. **(F)** Myocardial apoptosis in DCM mice–Western blot analysis of apoptosis-related proteins.

### 3.10 Drp1 overexpression abolishes the protection conferred by PIK3R1 deficiency against cardiac dysfunction and cardiomyocyte apoptosis in DCM mice *in vivo*

PIK3R1KO DCM mice were intervened with AAV-Drp1 for Drp1 overexpression to further verify the downstream regulatory mechanism of PIK3R1, and the experimental grouping was schematized (Figure 10A). Echocardiographic results showed that compared with WT-CON, WT-DCM mice had significantly decreased cardiac function; PIK3R1KO-DCM mice had significantly improved cardiac function compared with WT-DCM mice; Drp1 overexpression (PIK3R1KO-Drp1 OE) significantly reversed the improvement of cardiac function in PIK3R1KO-DCM mice, and there was no significant difference between PIK3R1KO-vector and PIK3R1KO-DCM groups (Figure 10B). HE staining, Masson’s trichrome staining, and WGA staining showed that compared with WT-CON, WT-DCM mice had severe myocardial remodeling; PIK3R1KO-DCM mice had significantly alleviated myocardial remodeling compared with WT-DCM mice; Drp1 overexpression significantly aggravated myocardial remodeling in PIK3R1KO-DCM mice, and there was no significant difference between PIK3R1KO-vector and PIK3R1KO-DCM groups (Figure 10C). Biochemical detection of serum showed that compared with WT-CON, WT-DCM mice had significantly increased levels of CK, CK-MB, and LDH; PIK3R1KO-DCM mice had significantly decreased levels of these factors compared with WT-DCM mice; Drp1 overexpression significantly increased the levels of these injury factors in PIK3R1KO-DCM mice, and there was no significant difference between PIK3R1KO-vector and PIK3R1KO-DCM groups (Figure 10D). TUNEL assay showed that compared with WT-CON, WT-DCM mice had significantly increased cardiomyocyte apoptosis; PIK3R1KO-DCM mice had significantly decreased apoptosis compared with WT-DCM mice; Drp1 overexpression significantly increased apoptosis in PIK3R1KO-DCM mice, and there was no significant difference between PIK3R1KO-vector and PIK3R1KO-DCM groups (Figure 10E). Western blot detection of apoptosis-related proteins showed that the change trend of Bax and cleaved caspase3 was consistent with the apoptosis trend, and Bcl-2 showed the opposite trend (Figure 10F). These results showed that the protection conferred by PIK3R1 deficiency against cardiac dysfunction, myocardial remodelling, myocardial injury and cardiomyocyte apoptosis is abolished by Drp1 overexpression, placing Drp1 downstream of PIK3R1 for these endpoints. Mitochondrial and ferroptosis endpoints were not assessed in this cohort and are therefore not claimed here.

**FIGURE 10:**
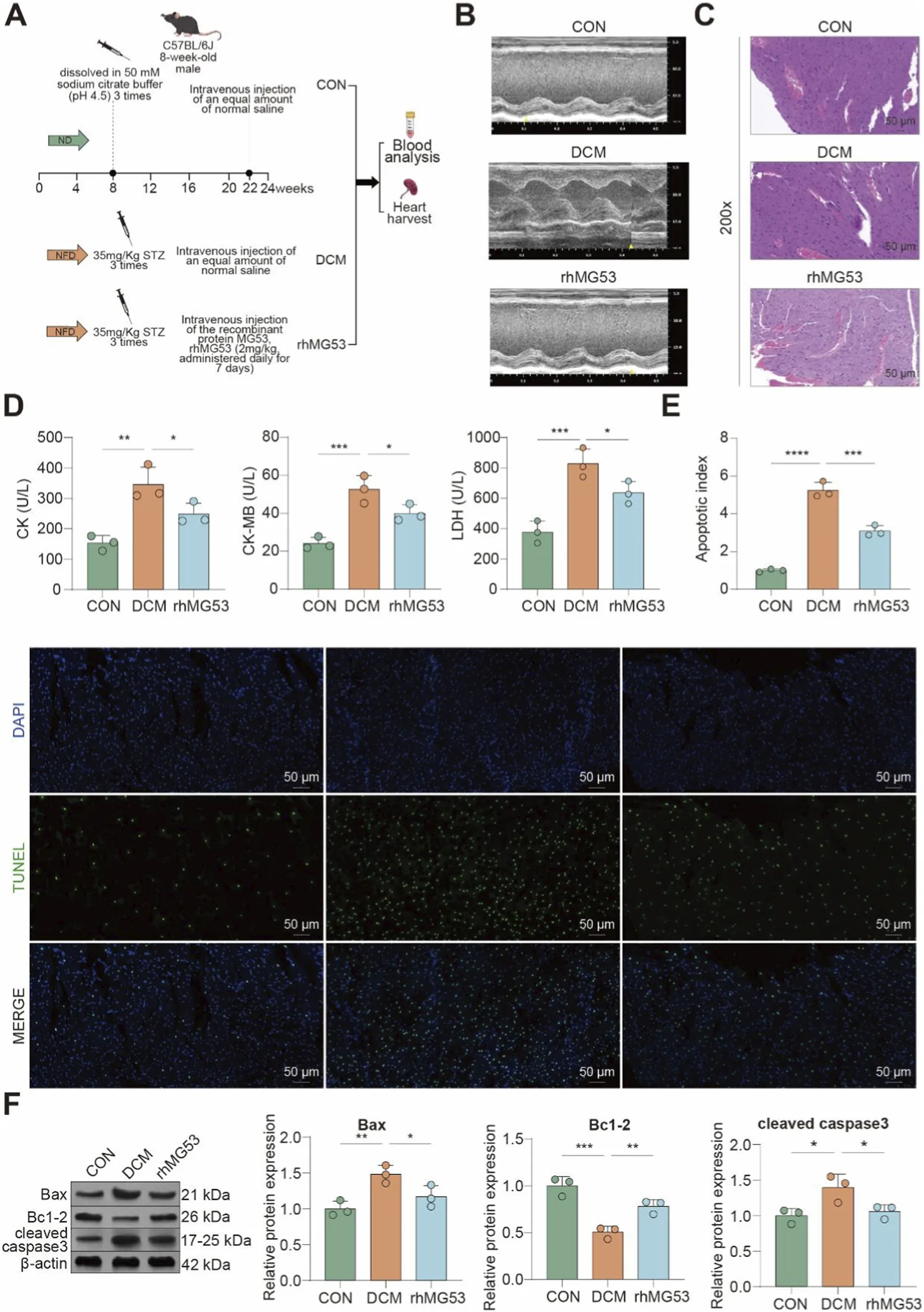
The mechanism by which PIK3R1 inhibition maintains mitochondrial homeostasis, suppresses cardiomyocyte ferroptosis and represses diabetic cardiomyopathy progression. **(A)** Schematic diagram of experimental grouping, showing modeling time nodes, interventions, experimental grouping, phenotypic identification methods, and tissue collection time points. **(B)** Cardiac function of DCM mice–echocardiographic assessment. **(C)** Cardiac function of DCM mice–HE staining of myocardial tissues. **(D)** Cardiac function indices of DCM mice–biochemical detection of myocardial function factors in serum. **(E)** Myocardial apoptosis in DCM mice–TUNEL assay for the detection of cardiomyocyte apoptosis. **(F)** Myocardial apoptosis in DCM mice–Western blot analysis of apoptosis-related proteins.

## 4 Discussion

Diabetic cardiomyopathy (DCM) is a progressive cardiovascular complication of diabetes mellitus, characterized by myocardial structural damage, dysfunction, and ultimately heart failure, which poses a significant threat to global public health (Zhang and Chen, 2012). The complex pathogenesis of DCM involves multiple interconnected mechanisms, including oxidative stress, mitochondrial dysfunction, cardiomyocyte apoptosis, and metabolic disorders (Capece et al., 2026). In recent years, ferroptosis, an iron-dependent form of regulated cell death driven by excessive lipid peroxidation, has emerged as a critical contributor to DCM progression (Dixon et al., 2012). However, the molecular regulators and signaling pathways governing ferroptosis in DCM remain incompletely elucidated. In this study, we systematically investigated the role of PIK3R1 in DCM using bioinformatics analysis, *in vivo* animal models, and multiple experimental techniques (Figures 1–10), and found that cardiomyocyte PIK3R1 deficiency is associated with preserved mitochondrial dynamic homeostasis, reduced cardiomyocyte ferroptosis and attenuated DCM progression, in parallel with increased PI3K/Akt/Nrf2 signalling.

Bioinformatics analysis is a powerful tool for identifying key genes involved in disease pathogenesis (Peng et al., 2023). Our study first screened DCM-related datasets and identified PIK3R1 as a significantly upregulated differentially expressed gene (Figure 1A). Verification in three independent external datasets confirmed the consistency of PIK3R1 overexpression in DCM (Figures 1B,C) KEGG enrichment analysis further showed that the PI3K-Akt signaling pathway was the most significantly enriched upregulated pathway, providing the rationale for examining this pathway in the subsequent experiments. These bioinformatics findings laid the foundation for subsequent *in vivo* experiments. We successfully established a DCM mouse model using HFD combined with low-dose STZ injection, as evidenced by impaired cardiac function and elevated blood glucose (Figures 2B,C). Importantly, we confirmed that PIK3R1 expression is significantly upregulated in the myocardial tissue of DCM mice at both the protein and mRNA levels (Figures 2E–G), which is consistent with the bioinformatics results. These data collectively suggest that PIK3R1 may be a key driver of DCM progression.

PIK3R1 encodes the p85α regulatory subunit of PI3K, which negatively regulates PI3K/Akt signaling by binding to the p110 catalytic subunit (Zhou et al., 2019). Accumulating evidence has shown that PIK3R1 is involved in the pathogenesis of various cardiovascular diseases, including heart failure and myocardial infarction (van Deel et al., 2025; Ma et al., 2022). However, its role in DCM remains unclear. To address this gap, we generated cardiomyocyte-specific PIK3R1KO mice and found that PIK3R1 deficiency significantly improves cardiac function in DCM mice, as demonstrated by increased LVEF and LVFS, reduced myocardial hypertrophy, and improved myocardial tissue structure (Figures 9B,C). Additionally, PIK3R1 deficiency decreased the levels of cardiac injury markers (CK, CK-MB, LDH) and inhibited myocardial apoptosis (Figures 9D–F). These results indicate that PIK3R1 plays a pathogenic role in DCM, and its deletion exerts a protective effect on the heart. Consistent with our findings, previous studies have shown that PIK3R1 deficiency protects against pressure overload-induced cardiac hypertrophy and myocardial ischemia-reperfusion injury (Azim et al., 2025; Syed et al., 2024), highlighting the potential of PIK3R1 as a therapeutic target for cardiovascular diseases.

Ferroptosis is characterized by iron accumulation, lipid peroxidation, and mitochondrial dysfunction (Stockwell et al., 2017). GPX4, a key antioxidant enzyme, inhibits ferroptosis by reducing lipid peroxides, while ACSL4 promotes ferroptosis by regulating lipid metabolism (Forcina and Dixon, 2019). Our study found that DCM mice exhibit typical features of ferroptosis, including increased myocardial iron deposition, downregulated GPX4 and SLC7A11 expression, and upregulated ACSL4 expression (Figures 5B,C,F). Notably, PIK3R1 deficiency significantly inhibits cardiomyocyte ferroptosis in DCM mice, as evidenced by reduced iron deposition, restored GPX4 and SLC7A11 expression, and decreased ACSL4 expression (Figures 8B,C,F). These results suggest that PIK3R1 deficiency is accompanied by reduced cardiomyocyte ferroptosis in DCM. Mitochondrial dysfunction is closely associated with ferroptosis, as mitochondria are both a major source of reactive oxygen species (ROS) and a target of lipid peroxidation (Zhou et al., 2020). Our transmission electron microscopy results showed that DCM mice have fragmented mitochondria with disrupted cristae, while PIK3R1 deficiency improves mitochondrial morphology (Figure 8A). Furthermore, PIK3R1 deficiency reduces mitochondrial ROS production (Figure 6C), indicating that the reduction in ferroptotic changes with PIK3R1 deficiency is accompanied by improved mitochondrial homeostasis. These findings are consistent with previous studies showing that mitochondrial dysfunction contributes to ferroptosis in various diseases (Gollmer et al., 2020; Liu et al., 2025). It should be emphasised that apoptosis was demonstrated just as consistently in the same hearts, by TUNEL staining and by the changes in Bax, Bcl-2 and cleaved caspase-3, and that ferroptotic and apoptotic indices were measured in parallel without any experiment designed to partition their relative contributions to myocardial injury. Necroptosis and pyroptosis were not examined, so the possibility that other regulated cell death pathways also operate in this model cannot be excluded.

The PI3K/Akt/Nrf2 pathway is a key signaling cascade involved in regulating antioxidant defense, mitochondrial function, and cell survival (Manning and Toker, 2017). Nrf2 is a master transcription factor that activates the expression of downstream target genes such as GPX4 and HO-1 to inhibit oxidative stress and ferroptosis (Zhong et al., 2024). Our study found that the PI3K/Akt/Nrf2 pathway is downregulated in DCM mice, as evidenced by decreased phosphorylation of PI3K and Akt, and reduced Nrf2 expression and nuclear translocation (Figures 7A–C). In contrast, PIK3R1 deficiency activates the PI3K/Akt/Nrf2 pathway and upregulates the expression of its downstream target genes (Figures 7A–D). These results indicate that PIK3R1 deficiency is associated with increased PI3K/Akt/Nrf2 signalling in DCM. Given that the PI3K/Akt pathway is a known upstream regulator of Nrf2 (Zhong et al., 2024), one interpretation of our findings is that PIK3R1 constrains Nrf2 through PI3K/Akt signalling; the present design cannot distinguish this from the alternative that the two changes occur in parallel, and pathway-specific inhibition would be required to separate them. This mechanism is supported by previous studies showing that activation of the PI3K/Akt/Nrf2 pathway attenuates ferroptosis in myocardial ischemia-reperfusion injury and diabetic nephropathy (Yang et al., 2025; Xia et al., 2025).

Mitochondrial dynamic homeostasis, including fission and fusion, is essential for maintaining mitochondrial function (Chen et al., 2023). Drp1 is a key regulator of mitochondrial fission, and its overexpression leads to mitochondrial fragmentation and dysfunction (Yu et al., 2026). Our study found that Drp1 overexpression reverses the protective effects of PIK3R1 deficiency on cardiac function and cardiomyocyte apoptosis in DCM mice (Figure 10). Specifically, Drp1 overexpression reduces LVEF and LVFS, aggravates myocardial remodelling, raises serum CK, CK-MB and LDH, and increases cardiomyocyte apoptosis (Figures 10B–F). These results indicate that Drp1 acts downstream of PIK3R1 for cardiac function and cardiomyocyte apoptosis; whether it also mediates the mitochondrial and ferroptotic effects was not tested in the rescue cohort. Consistent with our findings, previous studies have shown that Drp1-mediated mitochondrial fission contributes to ferroptosis in cardiomyocytes during myocardial ischemia-reperfusion injury (Liu et al., 2025). Moreover, we found that treatment with Mdivi-1, a specific inhibitor of mitochondrial fission, improves mitochondrial homeostasis, inhibits ferroptosis, and reduces cardiac injury in DCM mice (Figures 4, 5). This further confirms the importance of mitochondrial dynamic homeostasis in DCM progression and suggests that targeting mitochondrial fission may be a potential therapeutic strategy for DCM.

In addition to the novel mechanism uncovered, our study also has several clinical implications. First, myocardial PIK3R1 was consistently upregulated in DCM mice and in public mouse datasets (Figures 1A–C, 2E–G); whether PIK3R1 has any diagnostic value in patients cannot be addressed here, since it was not measured in any accessible clinical specimen. Second, the protection conferred by cardiomyocyte PIK3R1 deletion raises the possibility that PIK3R1 could be evaluated as a therapeutic target in DCM, a possibility that would first require confirmation in human tissue and in a pharmacological rather than genetic setting. Third, because Drp1 overexpression abolished that protection, combined inhibition of PIK3R1 and mitochondrial fission is a hypothesis worth testing in future work rather than a strategy supported by the present data. However, further clinical studies are needed to validate these potential applications.

This study has important limitations. No *in vitro* work in cultured cardiomyocytes was performed, so cell autonomy is not established, and no human myocardium was examined. Most importantly, no pathway-specific loss-of-function experiment was carried out; inhibition of PI3K, Akt or Nrf2 in PIK3R1-deficient DCM mice would be needed to test dependence, and until then the axis should be regarded as accompanying rather than mediating the protection, with Drp1 the only step tested directly. Consistent with this, nuclear Nrf2 was assessed by Western blotting with HO-1 as a downstream readout, without DNA-binding or ARE-reporter assays, so transcriptional activation remains an inference, and deletion efficiency was likewise inferred from de-repression of PI3K/Akt signalling rather than quantified in cardiac tissue. Mitochondrial fission was inhibited only pharmacologically, so off-target actions of Mdivi-1 cannot be excluded, and mitochondrial function was characterised by ATP, mitochondrial DNA copy number, reactive oxygen species and ultrastructure, without membrane potential, respiratory chain activity or extracellular flux analysis. Ferroptosis was assessed through iron, lipid peroxidation, GPX4, SLC7A11 and ACSL4, without FTH1, TFRC, PTGS2, 4-HNE or direct mitochondrial lipid reactive oxygen species. Finally, only male mice were used, so sex was not addressed as a biological variable; endpoints were confined to a single time point with no long-term outcome assessment; effects on fibroblasts and endothelial cells were not examined; and group size followed established practice in comparable models rather than an *a priori* power calculation.

In conclusion, our study systematically investigated the role and mechanism of PIK3R1 in DCM using bioinformatics analysis and *in vivo* experiments (Figures 1–10). We demonstrated that PIK3R1 is upregulated in DCM, and its deficiency improves cardiac function, inhibits cardiomyocyte ferroptosis, and ameliorates mitochondrial dysfunction via activating the PI3K/Akt/Nrf2 axis. Moreover, Drp1 overexpression reverses the protective effects of PIK3R1 deficiency, and inhibition of mitochondrial fission with Mdivi-1 exerts a protective effect on DCM mice. These findings indicate that cardiomyocyte PIK3R1 deficiency attenuates mitochondrial dynamic imbalance and ferroptotic changes in DCM mice in a Drp1-dependent manner, and that PI3K/Akt/Nrf2 signalling is a candidate mediator that requires direct confirmation.

## Data Availability

The original contributions presented in the study are included in the article/Supplementary Material, further inquiries can be directed to the corresponding author.
