# Supplementary material for: Cardiomyocyte PIK3R1 deficiency attenuates mitochondrial dynamic imbalance and ferroptosis in diabetic cardiomyopathy in association with the PI3K/Akt/Nrf2 axis
Source: Front Cell Dev Biol. 2026 Oct 21;14:1953037. doi: 10.3389/fcell.2026.1953037 (PMC13731403; doi:10.3389/fcell.2026.1953037)
Supplement: Supplementary file 1 [file Table1.docx]

**Supplementary Table 1 Primer sequence**

| **Gene** | **Primer** | **Sequence 5'-3'** |
| --- | --- | --- |
| β-actin | Forward Primer | 5'-GGCTGTATTCCCCTCCATCG-3' |
|  | Reverse Primer | 5'-CCAGTTGGTAACAATGCCATGT-3' |
| PIK3R1 | Forward Primer | 5'-GCTGGCTTTGGTGAAGATGA-3' |
|  | Reverse Primer | 5'-TCCAGGGTCACACTTCACCT-3' |
