# Supplementary material for: Cardiomyocyte PIK3R1 deficiency attenuates mitochondrial dynamic imbalance and ferroptosis in diabetic cardiomyopathy in association with the PI3K/Akt/Nrf2 axis
Source: Front Cell Dev Biol. 2026 Oct 21;14:1953037. doi: 10.3389/fcell.2026.1953037 (PMC13731403; doi:10.3389/fcell.2026.1953037)
Supplement: Supplementary file 2 [file Table2.docx]

**Supplementary Table 2 Antibody information**

| **Antibody** | **Manufacturer** | **Code** |
| --- | --- | --- |
| Anti-Bax | abcam | ab32503 |
| Anti-Bcl-2 | abcam | ab194583 |
| Cleaved Caspase 3 Polyclonal antibody | Proteintech | 25128-1-AP |
| Anti-Glutathione Peroxidase 4 | abcam | ab125066 |
| Anti-xCT | abcam | ab307601 |
| Anti-FACL4 | abcam | ab155282 |
| Anti-GAPDH | abcam | ab8245 |
| Anti-beta Actin | abcam | ab8226 |
| Goat Anti-Mouse IgG | Seville Biology | GB23301 |
| Goat Anti-Rabbit IgG | Seville Biology | GB23303 |
